# Transarterial chemoembolization combined donafenib with/without PD-1 for unresectable HCC in a multicenter retrospective study

**DOI:** 10.3389/fimmu.2023.1277329

**Published:** 2023-11-27

**Authors:** Hao Li, Jiacheng Wang, Guokun Zhang, Donglin Kuang, Yanliang Li, Xiang He, Cheng Xing, Yong Wang, Ming Shi, Xinwei Han, Jianzhuang Ren, Xuhua Duan

**Affiliations:** ^1^ Department of Interventional Radiology, The First Affiliated Hospital of Zhengzhou University, Zhengzhou, Henan, China; ^2^ Department of Hepatobiliary Surgery, The First Affiliated Hospital of Zhengzhou University, Zhengzhou, Zhengzhou, Henan, China; ^3^ Department of Interventional and Oncology, Dengzhou People's Hospital, Nanyang, Henan, China; ^4^ Department of Medical Imaging, Huaihe Hospital of Henan University, Kaifeng, Henan, China; ^5^ Department of Interventional Radiology, Zhoukou Central Hospital, Zhoukou, Henan, China; ^6^ Department of Interventional Vascular Surgery, The Second Affiliated Hospital of Hainan Medical University, Haikou, Hainan, China; ^7^ Department of Radiology, The Second Hospital of Xingtai, Xingtai, Hebei, China

**Keywords:** donafenib, programmed death-1 inhibitors, transarterial chemoembolization, unresectable hepatocellular carcinoma, multicenter retrospective study

## Abstract

**Background & aims:**

This multicenter retrospective study evaluated the efficacy and safety of transarterial chemoembolization (TACE) combined with donafenib and a programmed death-1 (PD-1) inhibitor (TACE+DP) and TACE combined with donafenib (TACE+D) for unresectable hepatocellular carcinoma (uHCC).

**Methods:**

The clinical data of 388 patients with uHCC who received TACE+DP or TACE+D as first-line treatment at six Chinese academic centers from July 2021 to July 2022 were collected and analyzed retrospectively. Patients in the TACE+DP group received an intravenous administration of a PD-1 inhibitor every three weeks and oral donafenib (0.2 g) twice daily until intolerable toxicity or disease progression. Patients in the TACE+D group received the same dose of donafenib for 3–5 days after TACE. Overall survival (OS) and progression-free survival (PFS)were analyzed by Kaplan-Meier method and log-rank test. The tumor response was compared between the two groups according to modified RECIST criteria. Adverse events were also analyzed between the two groups

**Results:**

The TACE+D group included 157 patients and the TACE+DP group included 166 patients. Patients in the TACE+DP group had a longer median OS (18.1 vs. 13.2 months, P<0.001) and longer median PFS (10.6 vs. 7.9 months, P<0.001) than those in the TACE+D group. Patients in the TACE+DP group achieved a greater objective response rate (ORR; 50.6% vs. 41.4%, P=0.019) and greater disease control rate (DCR) (89.2% vs. 82.8%, P=0.010) than those in the TACE+D group. No significant differences were found in the incidence or severity of adverse events between the TACE+DP and TACE+D groups (any grade: 92.9% vs. 94.6%, P=0.270; grade 3 or 4: 33.8% vs. 37.3%, P=0.253).

**Conclusion:**

With favorable safety and tolerability, TACE combined with donafenib and PD-1 inhibitors significantly improved PFS, OS, and ORR compared to TACE combined with donafenib.

## Introduction

Primary liver cancer is the sixth most common cancer and the fourth leading cause of cancer-related deaths worldwide; however, it remains a global health challenge, and its incidence is increasing (1). Hepatocellular carcinoma (HCC) is the leading histological form of primary liver cancer, representing 75–85% of cases (1). Given the tremendous heterogeneity of HCC, the patients’ performance levels, extent of underlying liver cirrhosis, and tumor burden influence the choice of the treatment plan (2). Surgical resection, ablation, and liver transplantation may provide curative potential for early-stage HCC. However, transarterial chemoembolization (TACE) is the main treatment for patients with intermediate-stage and advanced-stage HCC unsuitable for or refused to the curative approaches (3, 4).

Donafenib is a modified form of sorafenib with a trideuterated N-methyl group that enhances its molecular stability and improves its pharmacokinetic properties (5, 6). Simultaneously, the reduction in metabolites improves safety and tolerance. A phase 1 clinical trial of 300 patients evaluated the safety, tolerability, food effects, pharmacodynamics, and pharmacokinetics of donafenib in patients with advanced solid tumors. The oral donafenib was tolerated well, with manageable adverse events (AEs) (6). An open-label, randomized, parallel-controlled, phase II/III clinical study of donafenib as a first-line treatment for advanced HCC (ZGDH3) enrolled 668 patients with advanced HCC and found that overall survival (OS) was significantly longer in the donafenib group than in the sorafenib group (12.1 months vs. 10.3 months). In addition, the AE profiles were similar in both groups, with the sorafenib group being better tolerated than the sorafenib group. Donafenib is also the only monotherapy agent superior to sorafenib in first-line head-to-head studies of advanced HCC (7). Donafenib was first approved in China for treating patients with unresectable HCC (uHCC) who had not previously received systemic treatment (8). Like sorafenib, lenvatinib, and atezolizumab plus bevacizumab, donafenib is recommended as a first-line systemic treatment for patients with uHCC (9).

The first-line systemic therapies sorafinib (10) and lenvatinib (11) have demonstrated low objective response rates (ORRs) and limited benefits for OS. Studies have shown that TACE combined with molecularly targeted drugs has potential synergistic effects that prolong the OS of patients with uHCC (12, 13). The TACTICS (13) and LAUNCH (14) studies demonstrated that concurrent sorafenib and lenvatinib can delay tumor progression following TACE. However, TACE plus sorafenib did not show a significant OS benefit over TACE alone in the TACTICS trial for treating intermediate-stage HCC (15). Kudo et al. (15) demonstrated TACE plus sorafenib significantly improves progression-free survival (PFS) and clinically meaningful OS in patients with a high tumor burden in intermediate-stage HCC.

Currently, molecule-targeted drugs (16, 17) are combined with programmed cell death protein-1 (PD-1) inhibitors to improve PFS and OS in uHCC. TACE could cause an increase in VEGF and PD-L1 expression because of the hypoxic microenvironment after embolization. This effect is the theoretical basis of combining molecularly targeted drugs with PD-1 inhibitors and can be a promising complement to TACE (18). Some studies have focused on combining TACE with PD-1 and tyrosine kinase inhibitors (TKI) to treat uHCC (19, 20). To our knowledge, no similar study has evaluated TACE combined with donafenib and a PD-1 inhibitor to treat uHCC. Therefore, this real-world study explored the safety and efficacy of TACE combined with donafenib and a PD-1 inhibitor compared with donafenib alone in patients with uHCC.

## Materials and methods

### Patients

This study was approved by the ethics committee of our hospital and conducted in accordance with the Declaration of Helsinki. The requirement for written informed consent was waived because this study was retrospective. uHCC was confirmed by experienced hepatobiliary surgeons based on the National Comprehensive Cancer Network (NCCN) guidelines (21). From July 2021 to July 2022, 353 patients with uHCC who chose TACE combined with donafenib and a PD-1 inhibitor (TACE+DP) or TACE combined with donafenib (TACE+D) as the initial therapy were reviewed at six hospitals in China. These patients were classified into TACE+DP and TACE+D groups according to whether they received PD-1 inhibitor treatment.

The key eligibility criteria were 1) a diagnosis of uHCC confirmed by imaging and/or pathological diagnosis; 2) age 18–80 years with a life expectancy of at ≥3 months; (3) Barcelona Clinic Liver Cancer (BCLC) tumor stage B or C; 4) Eastern Cooperative Oncology Group (ECOG) performance status of 0–2 before TACE, and Child–Pugh class A or B7; 5) normal coagulation or renal function, corrected by appropriate treatment; 6) having received as least one cycle of systemic treatment (one dose of PD-1 inhibitor plus three weeks of donafenib at 200 mg twice daily) for the TACE+DP group, and three weeks of donafenib (200 mg twice daily) for the TACE+D group.

The exclusion criteria were 1) a tumor burden exceeding 70% of the whole liver, diffuse type HCC, or complete obstruction of the main portal vein by tumor thrombus; 2) having received previous systemic therapy, TACE, radioactive seed implantation, or intra-arterial chemoinfusion; 3) acceptance of conversion therapy, liver transplantation, intra-arterial chemoinfusion or other local–regional therapies (e.g., ablation, radioactive seed implantation) during the PFS period; 4) pulmonary fibrosis or autoimmune disease; 5) severe renal dysfunction, coagulation disorders, or cardiopulmonary dysfunction that cannot be corrected; 6) incomplete data.

### Treatment protocol

All patients underwent standardized conventional TACE (cTACE) or drug-eluting bead TACE (DEB-TACE) at each institution. For cTACE, an emulsion of 10–20 mL Lipiodol (Guerbet; Paris, France) was mixed with 20–40 mg doxorubicin or epirubicin and administered into the tumor-feeding vessels, followed by embolization with 150–350 μm or 350–560 μm polyvinyl alcohol particles or gelfoam particles until the tumor stain disappeared completely. For DEB-TACE, CalliSpheres (Jiangsu Hengrui Medicine Co., Ltd., Jiangsu, China) with 100–300 μm or 300–500 μm (loaded 40–60 mg doxorubicin or epirubicin) was used as described by Wang et al. (22). All TACE procedures were performed by physicians with at least 10 years of experience in interventional radiology at each participating center.

### Systemic therapy

Donafenib was administered orally for the first time 3 days after TACE at an initial dose of 200 mg twice daily. Patients in the TACE+DP group received intravenous administration of a PD-1 inhibitor (200 mg camrelizumab, 200 mg tislelizumab, or 240 mg toripalimab) every three weeks and 3 weeks with oral donafenib (200 mg twice daily). Patients in the TACE+D group received the same dose of donafenib 3–5 days after TACE. Donafenib was suspended 3 days before the subsequent TACE procedure. The PD-1 inhibitors and donafenib doses were reduced, suspended, or discontinued in patients who experienced AEs because of these agents. If donafenib-related adverse reactions are not tolerated, the dose should be adjusted to 200 mg/day.

### Assessment of treatment efficacy

Routine blood tests, liver and kidney functions, coagulation function, tumor markers, and enhanced MRI and/or CT were performed 4–6 weeks after the first TACE. Enhanced MRI and/or CT were used by two experienced radiologists to evaluate the best response according to the modified Response Evaluation Criteria in Solid Tumors (mRECIST) (23). The curative effects were evaluated using mRECIST, including complete response (CR), partial response (PR), stable disease (SD), and progressive disease (PD). ORR was defined as the ratio of CR+PR and the disease control rate (DCR) was defined as the proportion of CR+PR+SD. PFS was defined as the time interval from the first TACE procedure to the date of disease progression or death (23). OS was defined as the time from the first TACE procedure to death from any cause or the date of the last follow-up (23). TACE was repeated when the tumor still had an arterial blood supply as assessed by enhanced MRI and/or CT imaging, and a Child–Pugh classification of A or B was confirmed. AEs were graded using the Common Terminology Criteria for Adverse Events Version 4.0.

### Statistical methods

Quantitative data were calculated as means ± standard deviation and compared between the two groups using Student’s t-test. Categorical data were compared using the chi-squared or Fisher’s exact tests. Survival curves were estimated using Kaplan– Meier analysis and compared using log-rank tests. Factors affecting OS and PFS were predicted using univariate and multivariate Cox proportional hazard regression models and are described using the hazard ratio (HR) and confidence intervals (CI). Variables with p<0.1 in the univariate analysis were included in the multivariate analysis. Statistical significance was set at P<0.05. All data were analyzed using SPSS software (version 26.0; IBM Corporation; Armonk, NY, USA).

## Results

### Patient demographics

**Figure 1:**
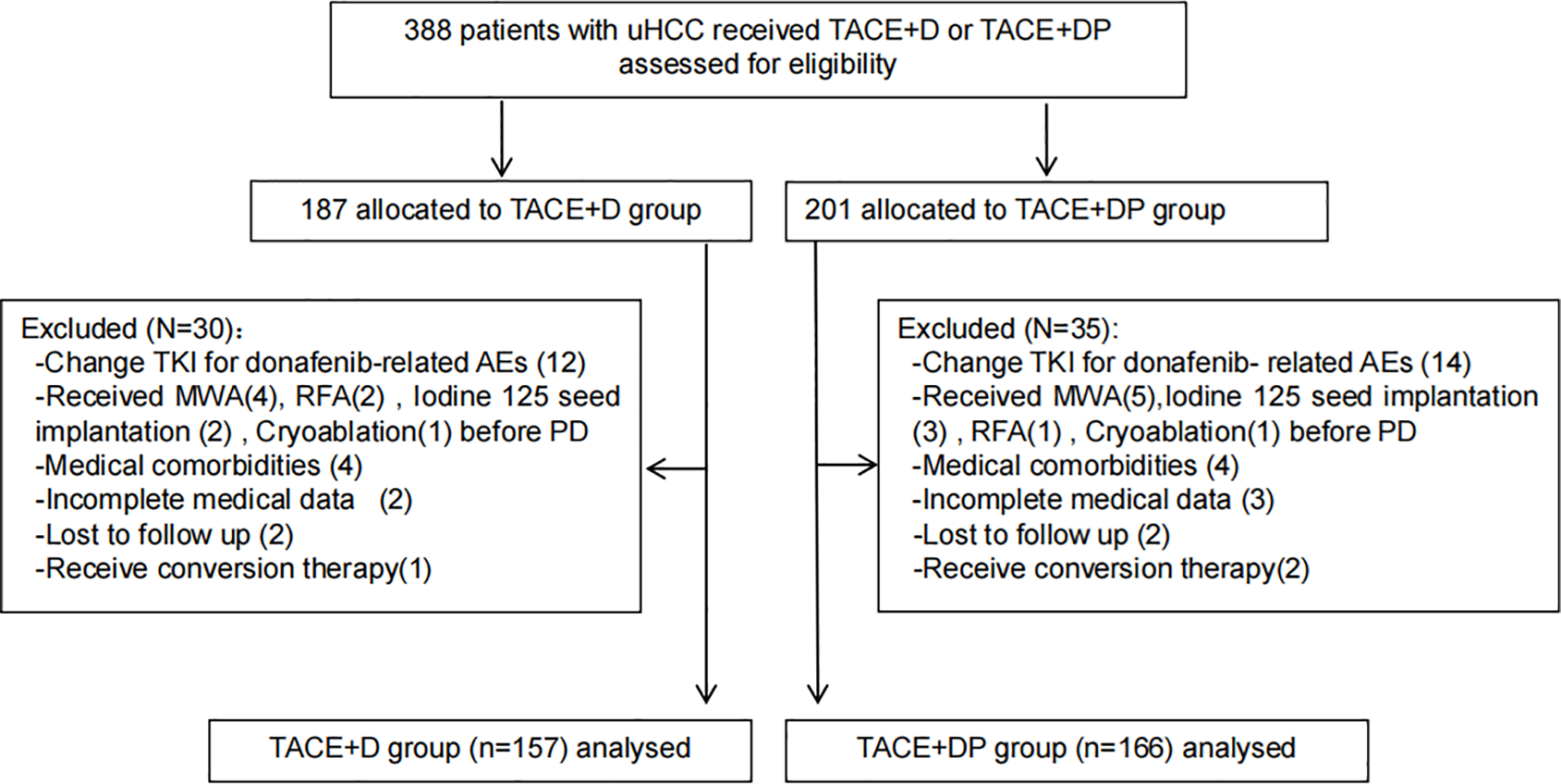
Patient flow chart. TACE+D, transarterial chemoembolisation combined with conbined with donafenib;TACE-A+C,transarterial chemoembolisation combined with donafenib and programmed death-1 (PD-1) inhibitor; TKI, tyrosine kinase inhibitors; AEs, adverse events; RFA, radiofrequency ablation; MWA, microwave Ablation; PD, progression disease.

From July 1, 2021, to July 1, 2022, 388 patients with uHCC who chose TACE+DP and TACE+D as the initial therapy were reviewed at six hospitals in China. Among these, 30 and 35 patients in the TACE+D and TACE+DP groups, respectively, were excluded. Ultimately, 323 patients were included in the analysis (157 in the TACE+D group and 166 in the TACE+DP group) (**Figure 1**). Baseline characteristics were well balanced between the two groups, with no statistically significant differences (**Table 1**). The institutional distribution was in **Supplementary Table 1**. The follow-up period ended on June 30, 2023.

**Table 1:** Patient characteristics at baseline.

Characteristics	TACE+D group (n=157)	TACE+DP group (n=166)	*P* value
Age (years), median (range)	54 (25-79)	52 (26-75)	0.828
Age group (years) (%)			0.249
<65	130 (82.8)	146 (88.0)	
≥65	27 (17.2)	20 (12.0)	
Sex (%)			0.602
Male	133 (84.7)	136 (81.9)	
Female	24 (15.3)	30 (18.1)	
ECOG PS (%)			0.373
0	66 (42.0)	79 (47.6)	
1	91 (58.0)	87 (52.4)	
Number of lesions, n (%)			0.622
≤3	61 (38.9)	70 (42.2)	
>3	96 (61.1)	96 (57.8)	
Portal vein invasion, n (%)			0.254
None	27 (17.2)	18 (10.8)	
Vp1-2	70 (44.6)	81 (48.8)	
Vp3-4	60 (38.2)	67 (40.4)	
Hepatic vein invasion, n (%)	30 (19.1)	29 (17.5)	0.813
Extrahepatic metastasis, n (%)	29 (18.5)	26 (15.7)	0.601
Child-Pugh class (%)			0.998
A	123 (78.3)	129 (77.7)	
B	34 (21.7)	37 (22.3)	
BCLC stage (%)			0.264
B	28 (17.8)	39 (23.5)	
C	129 (82.2)	127 (76.5)	
AFP (ng/mL) (%)			0.691
≤400	63 (40.1)	62 (37.3)	
>400	94 (59.9)	104 (62.7)	
Aetiology (%)			0.177
Hepatitis B	134 (85.4)	145 (87.3)	
Hepatitis C	16 (10.2)	9 (5.4)	
Non-B Non-C	7 (4.5)	12 (7.2)	
Tumor distribution (%)			0.579
Single	22 (14.0)	28 (16.9)	
Multiple	135 (86.0)	138 (83.1)	
maximum tumor diameter (cm, mean ± SD) (%)	8.6 ± 4.4	8.9 ± 4.9	0.563
<10cm	92 (58.6)	101 (60.8)	0.766
≥10cm	65 (41.4)	65 (39.2)	
No. of TACE no.(%)	3.14 ± 1.78	3.46 ± 1.35	0.071
1-2	56 (35.7)	63 (38.0)	0.757
≥3	101 (64.3)	103 (62.0)	
Duration of donafenib (months)			0.119
≤3months	49 (31.2)	38 (22.9)	
>3months	108 (68.8)	128 (77.1)	
Cycles of DP, n (%)			NA
≤4	–	62 (37.3)	
>4	–	104(62.7)	

TACE+D, transcatheter arterial chemoembolization (TACE) conbined

donafenib; TACE+DP, TACE conbined donafenib with programmed death-1 (PD-1) inhibitor; ECOG PS, Eastern Cooperative Oncology Group Performance Status; BCLC, Barcelona Clinic Liver Cancer; AFP, alpha-fetoprotein.

### Effectiveness analysis

**Figure 2:**
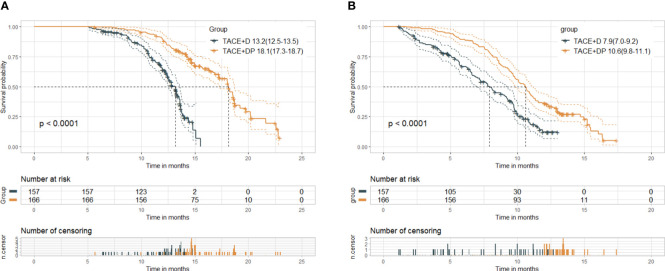
Kaplan-Meier curves of overall survival **(A)** and progression-free survival **(B)** in both groups. Median(95%CI). TACE+D, TACE plus donafenib; TACE+DP, TACE plus donafenib plus programmed death-1 (PD-1) inhibitor; TACE, transcatheter arterial chemoembolization; P value for log-rank test.

At the cutoff date (July 1, 2023), the median follow-up time was 14.6 months. Median follow-up was 17.4 (95%CI 16.7-18.0) months in the TACE+DP group and 13.1 (95%CI 12.8-13.5) months in the TACE+D group. The best patient responses after the first TACE are presented in **Table 2**. Based on mRECIST, patients in the TACE+DP group achieved a greater ORR (50.6% vs. 41.4%, P=0.019) and a greater DCR (89.2% vs. 82.8%, P=0.010) than those in the TACE+D group. In addition, patients in the TACE+DP group had longer median OS (18.1 vs. 13.2 months, P<0.001) (95% CI 17.3–18.7 months vs. 95% CI 12.5–13.5 months) and longer median PFS (10.6 vs. 7.9 months, P<0.001) (95% CI 9.8–11.1 months vs. 95% CI 7.0–9.2 months) than those in the TACE+D group (**Figure 2**).

**Table 2:** Tumor response based on mRECIST after the first TACE between the two groups.

Tumor response, n (%)	TACE+D group (n=157)	TACE+DP group (n=166)	P value
CR	24 (15.3)	40 (24.1)	0.011
PR	41 (26.1)	44 (26.5)	
SD	65 (41.4)	64 (38.6)	
PD	27 (17.2)	18 (10.8)	
ORR (CR+PR)	65 (41.4)	84 (50.6)	0.019
DCR (CR+PR+SD)	130 (82.8)	148 (89.2)	0.010

mRECIST, modified Response Evaluation Criteria in Solid Tumors;

TACE+D, transcatheter arterial chemoembolization (TACE) conbined withdonafenib; TACE+DP, TACE conbined with donafenib and programmed death-1 (PD-1) inhibitor; CR, complete response; PR, partial response; SD, stable disease; PD, progressive disease; ORR, objective response rate; DCR, disease control rate.

**Figure 3:**
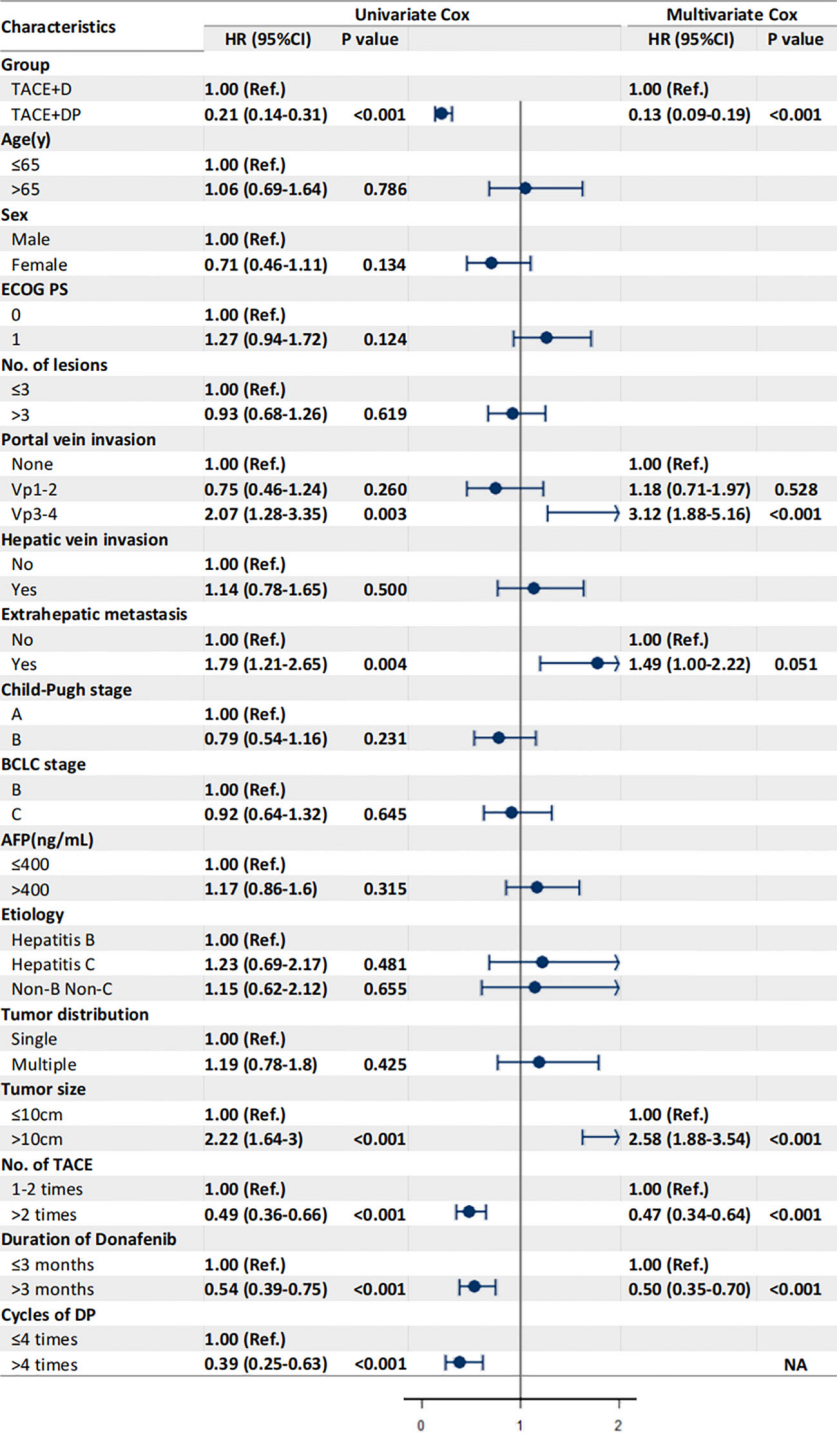
Univariate and multivariate analysis of prognostic factors associated with overall survival. Data are shown in HR (95%CI). ECOG, Eastern Cooperative Oncology Group Performance Status; Vp1, third branch portal vein invasion; Vp2, second branch portal vein invasion; Vp3, first branch portal vein invasion; Vp4, main portal vein invasion; AFP, alpha- fetoprotein; BCLC, Barcelona Clinic Liver Cancer; TACE, transcatheter arterial chemoembolization; NA, not applicable, variables not included in regression model.

**Figure 4:**
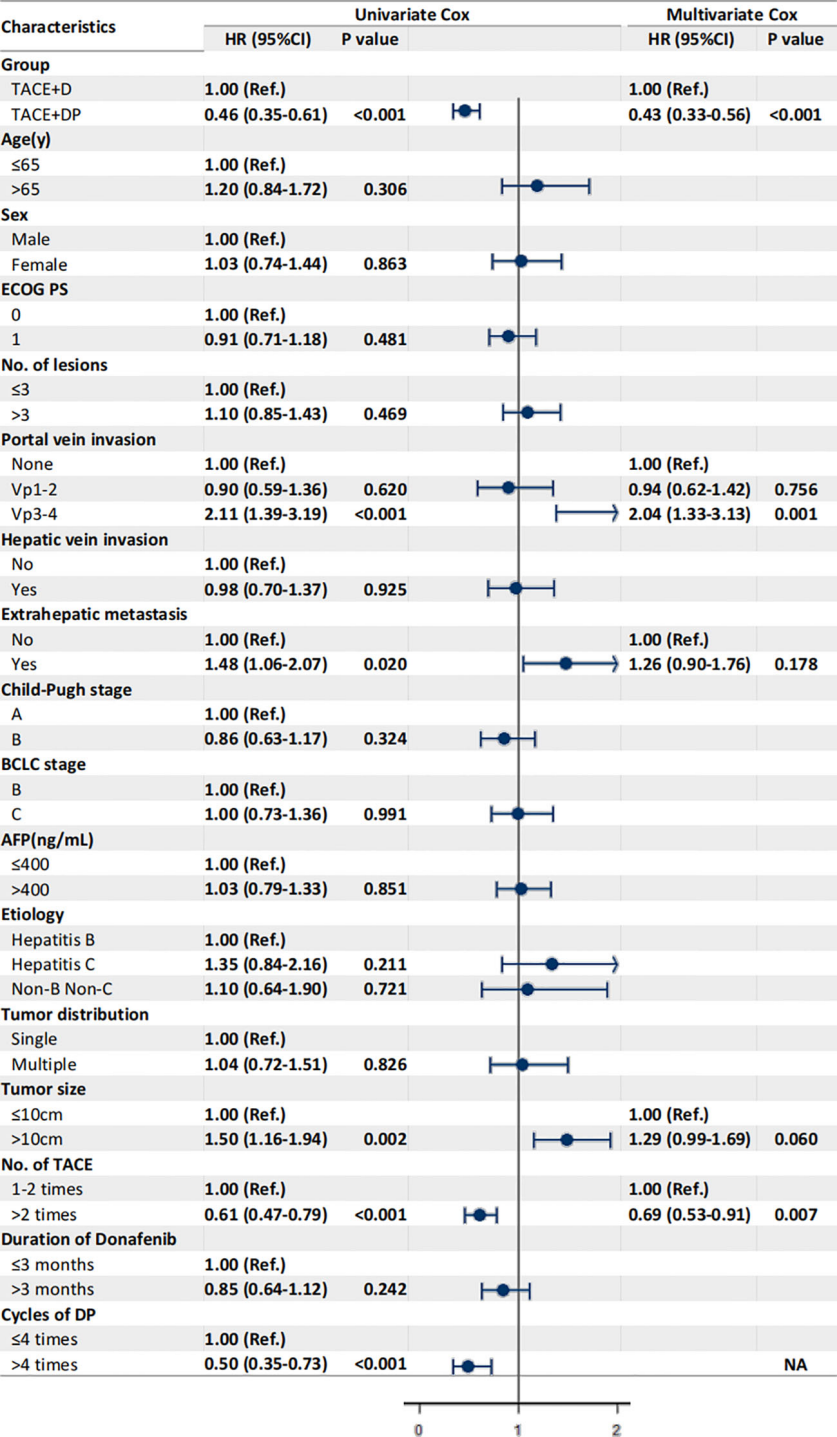
Univariate and multivariate analysis of prognostic factors associated with progression-free survival. Data are shown in HR (95%CI). ECOG, Eastern Cooperative Oncology Group Performance Status; Vp1, third branch portal vein invasion; Vp2, second branch portal vein invasion; Vp3, first branch portal vein invasion; Vp4, main portal vein invasion; AFP, alpha- fetoprotein; BCLC, Barcelona Clinic Liver Cancer; TACE, transcatheter arterial chemoembolization; NA, not applicable, variables not included in regression model.

**Figure 5:**
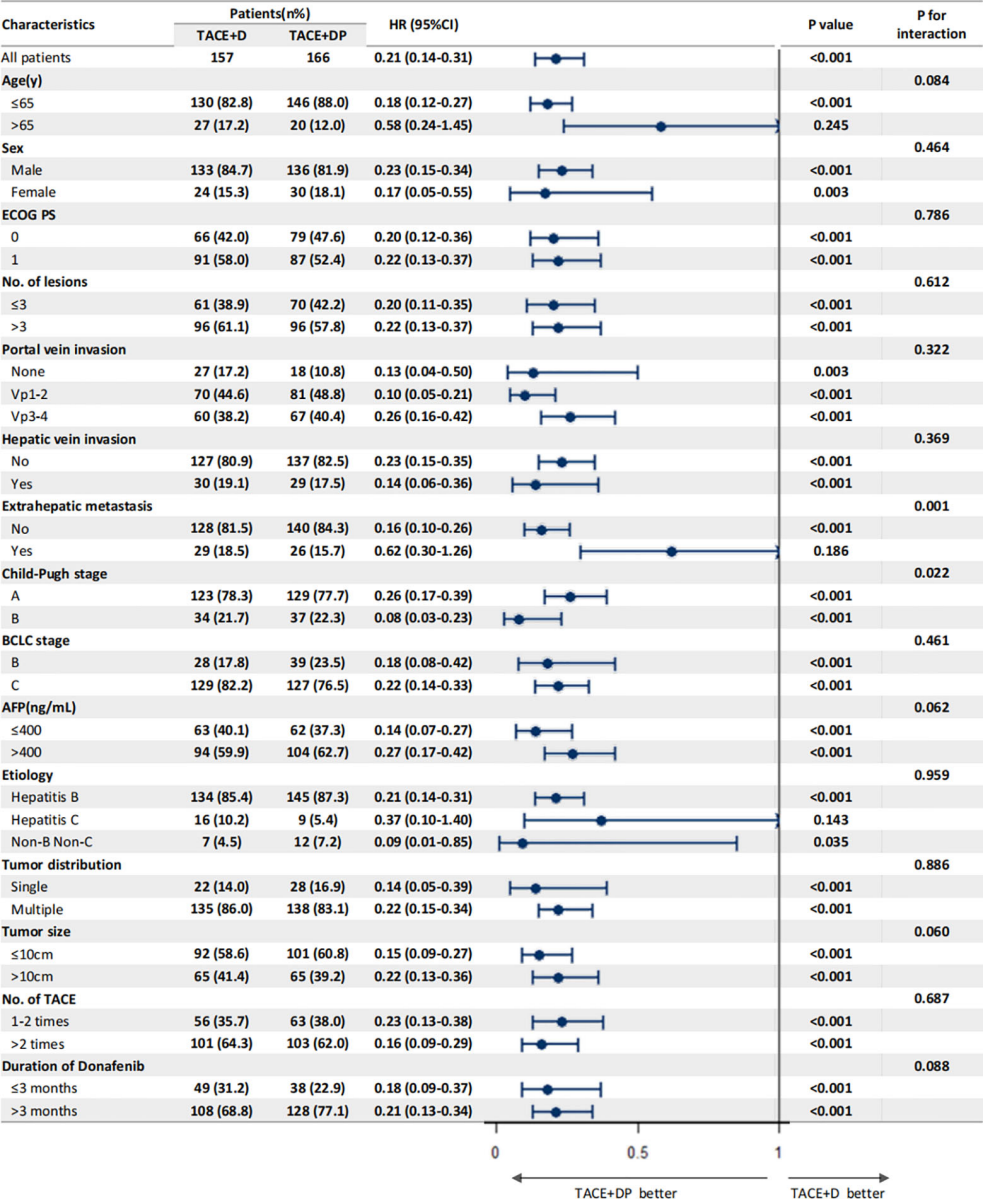
Subgroup analysis and forest plot of factors associated with OS in patients treated with TACE+D versus TACE+DP.

**Figure 6:**
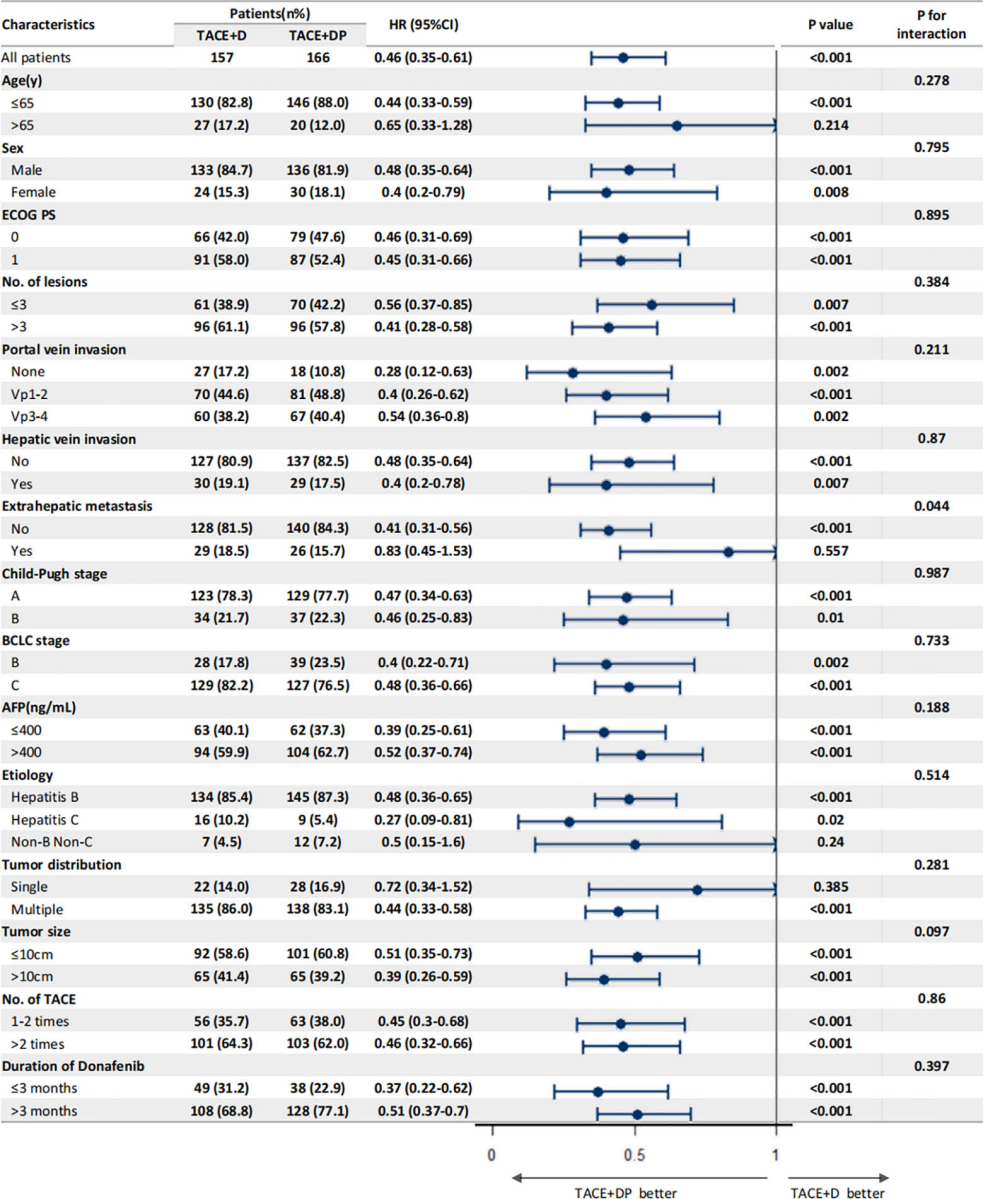
Subgroup analysis and forest plot of factors associated with PFS in patients treated with TACE+D versus TACE+DP.

The multivariable analysis identified that the independent prognostic factors for OS were patients in the TACE+DP group (HR=0.13, 95% CI 0.09–0.19, P<0.001), a maximum tumor diameter ≥10 cm (HR=2.58, 95% CI 1.88–3.54, P<0.001), portal vein invasion Vp3-4 (HR=3.12, 95% CI 1.88–5.16, P<0.001), having received >2 TACE (HR=0.47, 95% CI 0.34–0.64, P<0.001), and having received donafenib >3 months (HR=0.50, 95% CI 0.35–0.70, P<0.001) (**Figure 3**). In addition, PFS was independently associated with the patients in the TACE+DP group (HR=0.43, 95% CI 0.33–0.56, P<0.001), received >2 TACE (HR=0.69, 95% CI 0.53–0.91, P=0.007) and had portal vein invasion Vp3-4 (HR=2.04, 95% CI 1.33–3.13, P=0.001) (**Figure 4**). Subgroup analysis showed that TACE+DP group provided a better median OS and PFS over TACE+D group in almost patient subgroups (**Figures 5**, **6**).

### Safety

TACE-related AEs, including abdominal pain, nausea, vomiting, fever (post-embolization syndrome), ascites, and liver abscess, were not significantly different between the two groups (**Table 3**). The incidence and severity of AEs related to donafenib and/or PD-1 inhibitors in the TACE+DP group were comparable with the TACE+D group (any grade: 92.9% vs. 94.6%, P=0.270; grade 3 or 4: 33.8% vs. 37.3%, P=0.253) (**Table 3**). Because of grade 2/3 AEs, the dose of donafenib was reduced for 98 (45%) patients in the TACE+DP group and 76 (43%) patients in the TACE+D group and ultimately relieved. Reactive cutaneous capillary endothelial proliferation (RCCEP), hyperammonemia, and abdominal pain occurred more frequently in the TACE+DP group than in the TACE+D group (All P<0.05).

**Table 3:** Treatment related adverse events in the two groups.

Adverse events, n (%)	Any Grade			Grade 3 or 4		
	TACE+D group (n=157)	TACE+DP group (n=166)	P value	TACE+D group(n=157)	TACE+DP group (n=166)	P value
Adverse events related to TACE						
Fever	85 (54.1)	89 (53.6)	0.478	0	0	–
Pain	74 (47.1)	77 (46.4)	0.470	3 (1.9)	4 (2.4)	0.422
Gastrointestinal reaction	59 (37.6)	57 (34.3)	0.277	0	0	–
Nausea and vomiting	49(31.2)	51 (30.7)	0.478	0	0	–
Ascites	18 (11.5)	20 (12.0)	0.464	5 (3.2)	7 (4.2)	0.348
Liver abscess	1 (0.6)	2 (1.2)	0.345	0	2 (1.2)	0.129
Adverse events related to donafenib and/or programmed death-1 (PD-1) inhibitor						
Any adverse event	146 (92.9)	157 (94.6)	0.270	53 (33.8)	62 (37.3)	0.253
Hand-foot syndrome	76 (48.4)	80 (48.2)	0.484	15(7.6)	14 (8.4)	0.393
Diarrhea	44 (28.0)	49 (29.5)	0.421	9(5.7)	11 (6.6)	0.410
Hypertension	42(26.7)	50 (30.1)	0.256	15(9.5)	16(9.6)	0.484
Decreased platelet count	40(25.5)	48(28.9)	0.247	3(1.9)	5(3.0)	0.293
Fatigue	37 (23.5)	42 (25.3)	0.395	0	0	-
Abnormal liver function	33(21.0)	39 (23.5)	0.319	4(2.5)	6(3.6)	0.324
Alopecia	30(19.1)	35(21.1)	0.360	0	0	–
Rash	29(18.4)	34 (20.5)	0.355	0	0	-
Proteinuria	27(17.2)	32 (19.3)	0.343	1(0.6)	1(0.6)	0.484
Decreased appetite	22 (14.0)	28 (16.9)	0.245	0	0	-
Abdominal pain	5 (3.2)	12 (7.2)	0.043	1 (0.6)	2 (1.2)	0.345
Hyperammonemia	4 (2.5)	19 (11.4)	0.001	0	1(0.6)	-
Hypothyroidism	4 (0.9)	48 (10.6)	<0.001	0	0	-
Gastrointestinal hemorrhage	49 (10.7)	43 (9.5)	0.102	4(2.5)	3(1.8)	0.331
RCCEP	0	24(14.5)	–	0	1(0.6)	-
Immune-related pneumonia	0	4(2.4)	–	0	1(0.6)	-
Immune-related myocarditis	0	3(1.8)	–	0	0	-

TACE+D, transcatheter arterial chemoembolization (TACE) conbined withdonafenib; TACE+DP, TACE conbined with donafenib and programmed death-1 (PD-1) inhibitor; RCCEP, reactive cutaneous capillary endothelial proliferation.

Regarding donafenib and/or PD-1 inhibitor–related AEs, hypertension, hand-foot syndrome (HFSR), fatigue, and diarrhea were the most common AEs in both groups (**Table 3**). No donafenib or PD-1 inhibitor interruptions were observed, and 24 patients in the TACE+DP group who received camrelizumab developed RCCEP, a common skin toxicity caused by camrelizumab. Two patients (1.0%) were determined to have died from donafenib- and PD-1 inhibitor–related AEs, including one case of hepatic encephalopathy from donafenib and camrelizumab and one case of cerebral hemorrhage because of hypertension from donafenib and toripalimab. No grade 4 or 5 AEs occurred in the TACE+D group. None of the patients in the TACE+D group experienced AEs that resulted in death.

## Discussion

For patients with uHCC at BCLC stage B, TACE is strongly recommended, with the ORR beyond 50% (24). The TACTICS (13) and LAUNCH (14) studies demonstrated that TACE plus sorafenib or lenvatinib achieves greater tumor control rates than TACE alone. In a slight departure from the TACTICS study, LAUNCH included a greater proportion of patients with vascular invasion (71.8%) (15), finding that TACE plus lenvatinib achieved longer mOS (17.8 vs. 11.5 months), longer mPFS (10.6 vs. 6.4 months), and a greater ORR (54.1% vs. 25.0%, P <.001) than lenvatinib alone. TACE plus lenvatinib significantly improved ORR (53.1% vs. 25.0%) and OS (14.5 vs. 10.8 months) compared with plus sorafenib in patients with large tumor burden in BCLC C stage uHCC and with portal vein tumor thrombus (PVTT) (25). Combination therapy can improve outcomes in patients with uHCC and provide deeper and earlier disease control. The effectiveness of sorafenib or lenvatinib may also improve when the tumor burden is relatively low (3, 26, 27). In the retrospective study, TACE+D also improved to 7.9 months mPFS and 13.2 months mOS, demonstrating that donafenib could be a good substitute for sorafenib or lenvatinib in the combination therapy to improve the curative effect of uHCC.

Although the results of single-immune checkpoint inhibitor studies (KEYNOTE-240 and CheckMate 459) (28, 29) for advanced liver cancer have not met near-perfect expectations, their performance in the treatment of advanced liver cancer has been sufficient to focus on combination immunotherapy strategies. The success of the anti-VEGF plus PD-L1 dual blockade regimen in the first-line treatment of advanced hepatocellular carcinoma (IMbrave150 study) (30) marks an important change in the standard of care for advanced HCC. Despite the tumor heterogeneity of hepatocellular carcinoma in China and the West, combination therapy using TKI and PD-1 inhibitors currently shows great advantages for treating uHCC in China. The KEYNOTE-524 study reported that patients with locally advanced unresectable HCC treated with pembrolizumab plus lenvatinib achieved a 46.0% ORR, 9.3 months PFS, and 22 months mOS. The RESCUE study (31) showed that carrilizumab plus apatinib showed good antitumor activity and a manageable safety profile in both the first- and second-line treatment cohorts of patients with advanced HCC. In addition, the proportions of patients in the first- and second-line cohorts receiving interventional therapy were 62.9% and 77.5%, respectively. In addition, the primary endpoints of the LEAP-002 study (32), OS and PFS, did not achieve pre-specified statistical significance; however, subgroup analysis supported the combination regimen, with Finn specifically highlighting the high-risk profile of macrovascular invasion/extrahepatic spread (HR=0.78, 95% CI 0.63–0.95) and elevated alpha-fetoprotein(AFP) status (HR=0.67, 95% CI 0.50–0.90).

Thus, many studies have attempted to identify novel therapeutic strategies, such as TACE combined with TKI and PD-1 inhibitors, to improve the efficacy of uHCC treatment. However, related prospective randomized controlled trials, such as the LEAP-012 trial (NCT04246177) using lenvatinib plus pembrolizumab plus TACE vs. placebo in combination with TACE, have not reached the endpoint (33). In comparison, a prospective single-arm phase II clinical trial (TIDE) (34) of adjuvant tislelizumab plus donafenib in combination with TACE for postsurgical high-risk hepatocellular carcinoma did not meet the study endpoints. Retrospective studies have confirmed the effectiveness of TACE combined with TKI and PD-1 inhibitors for treating uHCC (18, 19). A systematic review of 15 retrospective studies showed that triple therapy with TACE, TKIs, and ICIs would provide a clinical benefit for uHCC in both short- and long-term outcomes without increasing severe AEs (33). The CHANCE001 study (35) results suggest that TACE plus PD-(L)1 blockade and molecule-targeted treatments could significantly improve PFS, OS, and ORR compared to TACE monotherapy for Chinese patients with predominantly advanced HCC in real-world practice, with an acceptable safety profile.

A single-center retrospective study found TACE combined with apatinib plus camrelizumab improved ORR from 17.3% to 42.9%, DCR from 57.7% to 85.7%, and OS from 13.1 to 24.8 months compared with apatinib plus camrelizumab (36). Another single-center retrospective study found TACE combined camrelizumab and sorafenib/lenvatinib resulted in 52.8% ORR, 81.1% DCR, and 8.5 months PFS (37). In a multicenter retrospective study of 62 patients treated with TACE plus lenvatinib and PD-1 inhibitors (38), 80.6% ORR was and 32 (53.2%) patients successfully converted to resectable HCC.

Donafenib has also been explored for the development of additional combination therapies. At an international conference in 2022, a phase Ib clinical study was the first to report the preliminary efficacy of donafenib combined with TACE and PD-1 inhibitors. The analysis found an 83.3% ORR in 12 patients with BCLC phase B, including three patients with CR (25.0%) and seven patients with PR (58.3%) (39). The donafinib combination therapy had a greater ORR than interventional therapy or drug therapy alone, no new safety risks, and was tolerated well.

In the current study, TACE+DP improved ORR from 41.4% to 50.6%, DCR from 82.8% to 89.2% vs. 82.8%, and PFS from 7.9 to 10.6 months compared with TACE+D. With the similar BCLC staging of tumors in the TACE combined with TKIs and PD-1 groups, we achieved a similar curative effect as Ju et al. (36). TACE combined with TKIs and ICIs may be promising therapeutic strategies for uHCC; their ORRs of 42.9–80.6% are better than the 33.2% ORR found in IMbrave150 (40). In our study, the improved efficacy of patients treated with TACE+DP may be due to the synergistic effects of TACE, donafenib, and PD-1 inhibitors. First, TACE caused tumor cell necrosis, effectively reduced tumor load, and activated immunogenic cell death (41). Second, similar to sorafenib, donafenib inhibits vascular endothelial growth factor and normalizes blood vessels. Finally, normalization of tumor vasculature can prompt immune cells to infiltrate the tumor area and enhance the immunocidal effect (42).

Notably, our subgroup analyses found that TACE+DP treatment was associated with favorable PFS versus TACE+D in patients with a significant tumor burden, such as portal vein invasion Vp3-4, maximum tumor diameter >10 cm, and three or more intrahepatic tumors, supporting the hypothesis that a low tumor burden after TACE might improve the efficacy of donafenib and PD-1 inhibitors. Multivariate analysis showed that patients in the TACE+DP group had independent protective factors for OS. In contrast, extrahepatic metastasis, three or more intrahepatic tumors, portal vein invasion Vp3-4, and a maximum tumor diameter >10 cm were independent risk factors. These results are consistent with previous studies on the risk factors of vascular invasion or extrahepatic metastases on the prognoses of patients with uHCC and suggest that assessing the tumor burden (e.g., number of tumors, distribution) becomes more important with the introduction of immunotherapy for uHCC, determining the timing and combination strategy of treatment with TACEs, TKIs, and ICIs.

The most common AEs of the triple combination modality were fever, abdominal pain, and liver function damage related to TACE (12, 13); hypertension, diarrhea, and HFSR related to TKIs (7, 13) and rash, fatigue, and pruritus related to PD-1 inhibitors (3, 24, 25). TACE combined with TKIs has been reported to increase AEs, including hypertension, HFSR, and diarrhea (26, 37). The most common AEs in the TACE+D group were HFSR, diarrhea, and hypertension, similar to those reported by Qin et al. (7) reported. TACE did not increase the AEs of donafenib because the donafenib dose reductions were applied for 45% of cases in the TACE+DP group and 43% of cases in the TACE+D group. In this study, AEs related to TACE+DP and TACE+D were similar to those reported above. The AEs of donafenib and/or PD-1 inhibitors were mild and manageable and did not lead to donafenib or PD-1 inhibitor interruptions.

The incidence rates of hyperammonemia, hypothyroidism, and RCCEP were greater in the TACE+DP group than in the TACE+D group. The blood–brain barrier can be damaged by the injury of brain endothelial cells by VEGFR receptor inhibitors (43). Hyperammonemia occurred in the TACE+DP group because of increased liver damage caused by the triple combination. Hypothyroidism is a common immune-related AE of PD-1 inhibitors (44). RCCEP is a common skin toxicity caused by camrelizumab that often occurs in patients treated with TACE+DP (45). The rate of RCCEP from TACE+DP treatment was dramatically lower than that of camrelizumab alone (21.5% vs. 66.8%), suggesting that the involvement of the VEGFA/VEGFR-2 signaling pathway in RCCEP pathogenesis (46) can be inhibited by donafenib.

This study had some limitations. First, this retrospective study had selection and indication biases, and the treatment option was determined based on the preference of the attending physician and the patient’s economic situation. Second, three PD-1 inhibitors were used, including camrelizumab, tislelizumab, and toripalimab. The bias of different PD-1 inhibitors was inevitable, and further subgroup studies with more cases for each PD-1 inhibitor should be performed. Third, two patients in the TACE+DP group and one patient in the TACE+D group who received conversion therapy were excluded from the study. Since the number of patients who received conversion therapy was relatively low, we did not perform further analyses. Fourth, the follow-up time was relatively short, and the number of research centers was small. Furthermore, a multicenter, prospective, controlled trial with a large sample size is required to confirm these preliminary results.

## Conclusion

In summary, TACE combined with donafenib and a PD-1 inhibitor demonstrated good clinical efficacy, durable antitumor responses, and significantly prolonged PFS and OS in patients with advanced HCC. Good tolerance to these combination therapies will enable further clinical studies. The prognoses of patients with more-advanced HCC are expected to improve through this novel and effective therapeutic strategy.

## Data availability statement

The raw data supporting the conclusions of this article will be made available by the authors, without undue reservation.

## Ethics statement

The studies involving humans were approved by The First Affiliated Hospital of Zhengzhou University. The studies were conducted in accordance with the local legislation and institutional requirements. Written informed consent for participation in this study was provided by the participants’ legal guardians/next of kin. Written informed consent was obtained from the individual(s) for the publication of any potentially identifiable images or data included in this article.

## Author contributions

XD: Methodology, Writing – review & editing. HL: Formal analysis, Writing – original draft. JW: Data curation, Resources, Writing – review & editing. GZ: Data curation, Resources, Writing – review & editing. DK: Formal analysis, Writing – review & editing. YL: Data curation, Resources, Writing – review & editing. XH: Data curation, Resources, Writing – review & editing. CX: Data curation, Resources, Writing – review & editing. YW: Data curation, Resources, Writing – review & editing. MS: Data curation, Resources, Writing – review & editing. XWH: Methodology, Resources, Writing – review & editing. JR: Methodology, Resources, Writing – review & editing.
