# Supplementary material for: Transarterial chemoembolization combined donafenib with/without PD-1 for unresectable HCC in a multicenter retrospective study
Source: Front Immunol. 2023 Nov 27;14:1277329. doi: 10.3389/fimmu.2023.1277329 (PMC10711098; doi:10.3389/fimmu.2023.1277329)
Supplement: Supplementary file 1 [file Table_1.docx]

Table S1. Study institutes.

| Study institutes. | Principal Investigators | Planned Number | Actual Number |
| --- | --- | --- | --- |
| The First Affiliated Hospital of Zhengzhou University | Xuhua Duan | 175 | 148 |
| Dengzhou People’s Hospital | Yanliang Li | 37 | 22 |
| Huaihe Hospital of Henan University | Xiang He | 54 | 31 |
| Zhoukou Central Hospital | Cheng Xing | 64 | 48 |
| The Second Affiliated Hospital of Hainan Medical University | Yong Wang | 52 | 38 |
| The Second Hospital of Xingtai | Ming Shi | 48 | 36 |
